# The adoption of generic drugs by a hospital: effects on drug dispensation among community pharmacies

**DOI:** 10.1186/s40780-018-0102-6

**Published:** 2018-03-14

**Authors:** Tomoya Tachi, Kosuke Saito, Hiroki Esaki, Ikuto Sugita, Aki Yoshida, Yuta Kanematsu, Yoshihiro Noguchi, Michi Umeda, Masahiro Yasuda, Takashi Mizui, Teruo Tsuchiya, Chitoshi Goto, Hitomi Teramachi

**Affiliations:** 1 Laboratory of Clinical Pharmacy, Gifu Pharmaceutical University, 1-25-4, Daigakunishi, Gifu-shi, Gifu, 501-1196 Japan; 2 Department of Pharmacy, Gifu Municipal Hospital, 7-1 Kashima-cho, Gifu-shi, Gifu, 500-8513 Japan; 3 Community Health Support and Research Center, 5-6-1 Kikuchi-Cho, Gifu-shi, Gifu, 500-8345 Japan; 4 Laboratory of Community Health Pharmacy, Gifu Pharmaceutical University, 1-25-4, Daigakunishi, Gifu-shi, Gifu, 501-1196 Japan

**Keywords:** Generic drug, Hospital, Community pharmacy, Adoption, Dispensation

## Abstract

**Background:**

The objective of the current study is to elucidate the effect that the adoption of generic drugs by a large hospital has on the dispensation of generic drugs by community pharmacies. We evaluated the percentage of generic drugs dispensed by pharmacies and the cost of drugs dispensed before and after the adoption of generic drugs by a large hospital.

**Methods:**

Participants comprised patients who were admitted to Gifu Municipal Hospital prior to its adoption of generic drugs (November 1, 2013 to November 14, 2013) and after its adoption (November 1, 2014 to November 14, 2014) and who utilized generic drugs dispensed by pharmacies.

**Results:**

Results indicated that the pre-adoption dispensation rate of generic drugs by pharmacies was 48.3% (477/926 drugs), while the post-adoption rate was 57.7% (604/1046 drugs), indicating an increase of 9.4 points (*P* < 0.001). Furthermore, an investigation into the price paid for generic drugs as a percentage of the total price paid for all drugs indicated the following: the pre-adoption percentage was 23.5% (9756/41,461 yen), and the post-adoption percentage was 34.1% (19,221/56,438 yen), indicating an increase of 10.6 points (*P* < 0.001).

**Conclusions:**

The results of this study revealed that the adoption of generic drugs by a hospital may promote the use of generic drugs by pharmacies and lead to reduced medical costs as well.

## Background

The use of generic drugs as a way to reduce medical costs has been increasing around the world. In 2015, the global percentage of generic drug use was reported to be high, ranging between 60% and 90%. In Japan, however, this percentage remains significantly lower, at 54.6% [1]. As Japanese society has continued to age, medical costs have also increased. For example, the national cost of medical care in Japan in 2015 was 4.1 billion yen [2], an amount that is putting a great deal of pressure on national health care financing. Thus, the Ministry of Health, Labour and Welfare of Japan drew up its “Roadmap to Further Promote the Use of Generics” in April 2013 in order to increase the use of generic drugs. This document specifies that “the numerical share of generic drugs is to be increased to at least 60% of all drugs by March 2018” [3]. In Japan, reforms of national medical policies and the medical system at both the national and local levels are being conducted in order to facilitate increased use of generic drugs, and medical facilities are also carrying out a variety of initiatives to this end. There are numerous reports creating basic resources for promoting the use of generic drugs, such as elucidation of patient-related factors and physician-related factors, and evaluation of healthcare policies and promotional activities, in various countries [4–15].

One of the reforms made to the national healthcare system was the 2014 revision of the reimbursement system for medical care. This revision ensures that hospitals that qualify for the diagnosis procedure combination system and have a high percentage of generic drug use among their in-hospital patients receive higher rates of reimbursement. Many hospitals that take advantage of this higher reimbursement system utilize a large number of generic drugs in their in-hospital pharmacies. In fact, it has been reported that the rate of generic drug usage by in-hospital patients has been increasing [16]. It has also been reported that the use of generic drugs by hospitals has led to increases in generic drug use by in-hospital patients and has been effective in decreasing in-hospital medical costs [17]. If a large hospital begins implementing generic drug use, this is likely to influence the dispensing of generic drugs at community pharmacies. However, there exists no research as to whether there is an effect, and if so what type, of the adoption of generic drugs within a hospital mainly for use on hospitalized patients on the percentage of non-hospital generic drug prescriptions filled by pharmacies, as well as on the cost of the drugs dispensed by these pharmacies. Such research is extremely important in order to create a policy designed to increase the widespread use of generic drugs.

In this study, we assessed the dispensation rate of generic drugs by pharmacies and the drug costs at pharmacies before and after the adoption of generic drugs by a large hospital in order to clarify its effect on the dispensation of generic drugs by community pharmacies. We also compared the price of drugs used within the hospital and the price of drugs dispensed by pharmacies.

## Methods

### Participants

**Fig. 1:**
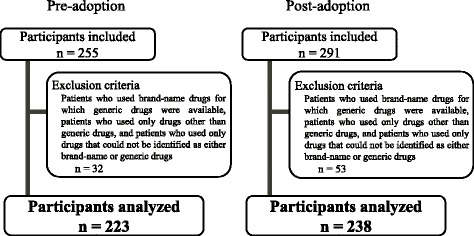
Patients included as participants

Participants comprised patients who utilized drugs dispensed by pharmacies and who were hospitalized at Gifu Municipal Hospital between November 1, 2013 and November 14, 2013, which was prior to its adoption of generic drugs (*n* = 255), and again between November 1, 2014 and November 14, 2014, which was after the adoption of generic drugs by the hospital (*n* = 291). The exclusion criteria were as follows: patients who used brand-name drugs for which generic drugs were available, patients who used only drugs other than generic drugs, and patients who used only drugs that could not be identified as either brand-name or generic. After applying the exclusion criteria, a total of 223 participants from the pre-adoption period and 238 participants from the post-adoption period were included in analyses (Fig. 1).

### Analyzed drugs and the situation of adoption of drugs in the hospital

The drugs we analyzed were “brand-name drugs with available generic drugs” and “generic drugs” for both internal and external use that were included in the calculation formula for the numerical share of generic drugs listed in the “Roadmap to Further Promote the Use of Generics” [3].

With a total of 609 beds, Gifu Municipal Hospital is one of the largest general hospitals in Japan, providing community medical services and emergency care to the city of Gifu and its suburbs. Gifu Municipal Hospital adopted a large number of generic drugs between March and May 2014. The percentage of generic drugs for internal or external use that were adopted at Gifu Municipal Hospital was 16.0% (60 /375 drugs) prior to the adoption of generic drugs and 32.0% (138/430) after adoption.

### Survey details

A retrospective survey of electronic medical records was conducted. The surveyed items were patient age, gender, medical department where hospitalized, type of medical insurance, and drugs utilized immediately before admission. Drugs used immediately before hospital admission were classified by prescribing facility (i.e., drugs prescribed by Gifu Municipal Hospital or the other hospitals/clinics) or dispensing facility (i.e., drugs dispensed by pharmacies or hospitals/clinics, including pharmacies within hospitals/clinics). They were also categorized as either brand-name drugs or generic drugs, and further categorized by drug price and efficacy.

### Assessment methods and statistical analysis

In order to assess the changes that occurred in the dispensation of generic drugs at pharmacies before and after the adoption of generic drugs by Gifu Municipal Hospital, we established the following outcome measures: the dispensation rate of generic drugs at hospitals before and after adoption (the percentage of the number of drugs) and the price paid for generic drugs (expressed as a percentage of the total price paid for all drugs, referred to here as the “price percentage”). In addition, in order to confirm whether the adoption of generic drugs by Gifu Municipal Hospital indeed had an effect on the dispensation rate of generic drugs, as well as the drug ratio, we performed two types of stratification: [1] stratification of drugs that had the same ingredients as the newly adopted generic drug vs. drugs that did not, and [2] stratification by prescribing facility.

In order to assess whether the dispensation of generic drugs by pharmacies led to reduced drug costs, we calculated the reduced price of drugs – which was the difference between the price of the drugs actually utilized (actual drug cost) and the cost of drugs dispensed at maximum price (drug cost at maximum price) – among drugs of the same type and specification, for each price per drug or per unit. Conversions of the unit of currency utilized in this study (Japanese yen) are as follows: 1 U.S. dollar = 110.2 Japanese yen, 1 euro = 129.8 Japanese yen (as of August 15, 2017).

In order to determine the amount of reduction in the costs of drugs utilized in Gifu Municipal Hospital and drugs dispensed by pharmacies, we assessed the price ranges of drugs utilized in the hospital and drugs dispensed by pharmacies after generic drugs were adopted. In the assessment, the targets were drugs prescribed by Gifu Municipal Hospital and dispensed by pharmacies that included the same ingredients as those used in the hospital. These were then divided into the following four groups based on the price ranges of drugs utilized in the hospital and drugs dispensed by pharmacies: [1] brand-name drugs with generics available, [2] generic drugs (50% or more of the price of the brand-name drugs or the most expensive generics), [3] generic drugs (between 30 to 50% of the price of the brand-name drugs or the most expensive generics), and [4] generic drugs (less than 30% of the price of the brand-name drugs or the most expensive generics). We investigated whether the price ranges of drugs dispensed by pharmacies were lower, higher, or the same as the price ranges of drugs utilized in the hospital. In Japan, there are three price ranges for all generic drugs already listed in the health insurance reimbursement system that have the same formulation, dosage form classification, and specifications [18]. Thus, there were four groups in this study, including the price range group for brand-name drugs (three price range groups for generics and one price range group for brand-name drugs). Statistical analysis was performed using IBM SPSS Statistics 22 (Armonk, New York). Comparisons of the dispensation rates and drug price rates before and after the adoption of generic drugs were performed using a chi-square test because the variables were unpaired despite involving a pre-post comparison. Statistical significance was set at *P* < 0.05.

### Ethical considerations

This study was approved by the Ethical Review Board of Gifu Municipal Hospital (Approval number: 212) and the Bioethics Committee of Gifu Pharmaceutical University (Approval number: 311–2) and was carried out according to Ethical Guidelines for Medical and Health Research Involving Human Subjects announced by the Ministry of Health, Labour and Welfare in Japan.

## Results

### Patient and drug background

Background information regarding the 223 pre-adoption period participants and the 238 post-adoption period participants is shown in Table 1. The drugs assessed in this study were 926 drugs utilized prior to the adoption of generic drugs and 1046 drugs utilized after adoption. Background information regarding all these drugs is shown in Table 2.

**Table 1:** Patient background

	Pre-adoption (*n* = 223)	Post-adoption (*n* = 238)	*P*
Age (years), mean ± standard deviation	68.5 ± 18.9	68.7 ± 17.4	0.911
Gender [n (%)]			0.608
Male	124 (55.6)	139 (58.4)	
Female	99 (44.4)	99 (41.6)	
Prescribing facility [n (%)]			0.470
Gifu Municipal Hospital	106 (47.5)	104 (43.7)	
Other hospitals/clinics	140 (62.8)	159 (66.8)	
Medical department [n (%)]			
Cardiovascular Medicine	45 (20.2)	41 (17.2)	
Neurology	2 (0.9)	3 (1.3)	
Nephrology	4 (1.8)	3 (1.3)	
Gastroenterology	29 (13.0)	27 (11.3)	
Respiratory and Medical Oncology	23 (10.3)	20 (8.4)	
Hematology	9 (4.0)	7 (2.9)	
General Internal Medicine	4 (1.8)	13 (5.5)	
Psychiatry	4 (1.8)	5 (2.1)	
Pediatrics	11 (4.9)	7 (2.9)	
Surgery	23 (10.3)	24 (10.1)	
Neurosurgery	2 (0.9)	1 (0.4)	
Breast Surgery	4 (1.8)	10 (4.2)	
Orthopedic Surgery	17 (7.6)	21 (8.8)	
Thoracic & Cardiac Surgery	6 (2.7)	6 (2.5)	
Obstetrics & Gynecology	6 (2.7)	5 (2.1)	
Ophthalmology	12 (5.4)	8 (3.4)	
Dermatology	4 (1.8)	4 (1.7)	
Urology	13 (5.8)	23 (9.7)	
Otolaryngology & Head and Neck Surgery	4 (1.8)	7 (2.9)	
Dental & Oral Surgery	1 (0.4)	3 (1.3)	
Type of public medical insurance [n (%)]			
Union-managed health insurance	11 (4.9)	9 (3.8)	
Kyokai Kenpo	29 (13.0)	26 (10.9)	
All mutual aid associations	7 (3.1)	6 (2.5)	
National Health Insurance	63 (28.3)	77 (32.4)	
Advance elderly	96 (43.0)	102 (42.9)	
Retired persons	2 (0.9)	2 (0.8)	
Tax-exempt out-of-pocket	0 (0.0)	0 (0.0)	
Livelihood Protection Law	15 (6.7)	16 (6.7)

**Table 2:** Drug background

	Pre-adoption (*n* = 926)	Post-adoption (*n* = 1046)	*P*
Drugs that have the same ingredients or different ingredients as the newly adopted generic drugs [n (%)]			0.255
Drugs with same ingredients	302 (32.6)	339 (32.4)	
Drugs with different ingredients	624 (67.4)	707 (67.6)	
Prescribing facility [n (%)]			< 0.001^*^
Gifu Municipal Hospital	415 (44.8)	382 (36.5)	
Other hospitals/clinics	511 (55.2)	664 (63.5)	
Internal/External use [n (%)]			0.304
Internal use drugs	844 (91.1)	938 (89.7)	
External use drugs	82 (8.9)	108 (10.3)	
Efficacy classification [n (%)]			
Central nervous system	111 (12.0)	162 (15.5)	
Peripheral nervous system	3 (0.3)	10 (1.0)	
Sensory organs	15 (1.6)	33 (3.2)	
Cardiovascular	262 (28.3)	299 (28.6)	
Respiratory	41 (4.4)	35 (3.3)	
Gastrointestinal	205 (22.1)	208 (19.9)	
Hormones	0 (0.0)	1 (0.1)	
Urogenital organs and anus	7 (0.8)	14 (1.3)	
Dermatologic preparations	40 (4.3)	48 (4.6)	
Vitamins	33 (3.6)	22 (2.1)	
Nutrients, tonics, and alternatives	10 (1.1)	14 (1.3)	
Blood and body fluids	83 (9.0)	71 (6.8)	
Other metabolic drugs	51 (5.5)	70 (6.7)	
Antineoplastic agents	4 (0.4)	7 (0.7)	
Allergy medicines	30 (3.2)	21 (2.0)	
Antibiotics	23 (2.5)	12 (1.1)	
Chemotherapeutics	7 (0.8)	16 (1.5)	
Alkaloidal narcotics	0 (0.0)	2 (0.2)	
Non-alkaloidal narcotics	1 (0.1)	1 (0.1)	

^*^P < 0.05

### Dispensation rate of generic drugs

The dispensation rates of generic drugs at pharmacies prior to and after adoption are shown in Table 3. The dispensation rate of generic drugs at pharmacies increased significantly by 9.4 points. Drugs with the same ingredients as the newly adopted generic drugs accounted for a significant increase of 19.3 points while drugs that did not have the same ingredients as the newly adopted generic drugs accounted for a non-significant increase of 4.7 points. Drugs prescribed by Gifu Municipal Hospital accounted for a significant increase of 19.0 points while drugs prescribed by other medical facilities accounted for a non-significant increase of 3.8 points.

**Table 3:** Dispensation rate of generic drugs by pharmacies

	Dispensation rate of generics		*P*
	Pre-adoption [% (drugs/total drugs)]	Post-adoption [% (drugs/total drugs)]	
All drugs subjected to analysis	48.3 (447/926)	57.7 (604/1046)	< 0.001^*^
Drugs that have the same ingredients or different ingredients as the newly adopted generic drugs			
Drugs with same ingredients	44.7 (135/302)	64.0 (217/339)	< 0.001^*^
Drugs with different ingredients	50.0 (312/624)	54.7 (387/707)	0.094
Priscribing facility			
Gifu Municipal Hospital	47.5 (197/415)	66.5 (254/382)	< 0.001^*^
Other hospitals/clinics	48.9 (250/511)	52.7 (350/664)	0.219

^*^P < 0.05

### Price percentage of generic drugs

The price percentages of generic drugs dispensed by pharmacies prior to and after adoption are shown in Table 4. The price percentage of generic drugs dispensed by pharmacies increased significantly by 10.6 points. Drugs with the same ingredients as the newly adopted generic drugs accounted for a significant increase of 19.8 points while drugs that did not have the same ingredients as the newly adopted generic drugs accounted for a significant increase of 7.7 points. Drugs prescribed by Gifu Municipal Hospital accounted for a significant increase of 12.9 points while drugs dispensed at other medical facilities accounted for significant increase of 8.2 points.

**Table 4:** Price percentage of generic drugs at pharmacies

	Price percentage of generic drugs		*P*
	Pre-adoption [% (yen/total yen)]	Post-adoption [% (yen/total yen)]	
All drugs subjected to analysis	23.5(9756/41,461)	34.1(19,221/56,438)	< 0.001^*^
Drugs that have the same ingredients or different ingredients as the newly adopted generic drugs			
Drugs with same ingredients	27.1(3828/14,144)	46.8(7113/15,207)	< 0.001^*^
Drugs with different ingredients	21.7(5928/27,318)	29.4(12,108/41,231)	< 0.001^*^
Priscribing facility			
Gifu Municipal Hospital	21.5(4517/20,975)	34.4(8059/23,455)	< 0.001^*^
Other hospitals/clinics	25.6(5240/20,487)	33.8(11,162/32,983)	< 0.001^*^

^*^P < 0.05

### Reduction in drug costs by price per drug or per unit

As part of our investigation into drugs dispensed by pharmacies, we calculated reductions in drug costs using price per drug or price per unit. Results indicated that the cost reduction was 9.9 yen per drug/unit prior to adoption, indicating a reduction of 18.2%, while the cost reduction was 17.0 yen per drug/unit after adoption, indicating a reduction of 24.0%.

For drugs prescribed by Gifu Municipal Hospital, the cost reduction was 9.2 yen per drug/unit prior to adoption, indicating a reduction of 15.3%. The cost reduction was 20.9 yen per drug/unit after adoption, indicating a reduction of 25.4%. For drugs prescribed at other medical facilities, the cost reduction was 10.6 yen per drug/unit prior to adoption, indicating a reduction of 20.9%. The cost reduction was 4.8 yen per drug/unit after adoption, indicating a reduction of 2.9%.

### Price difference between in-hospital use drugs and drugs dispensed by health insurance pharmacies

**Fig. 2:**
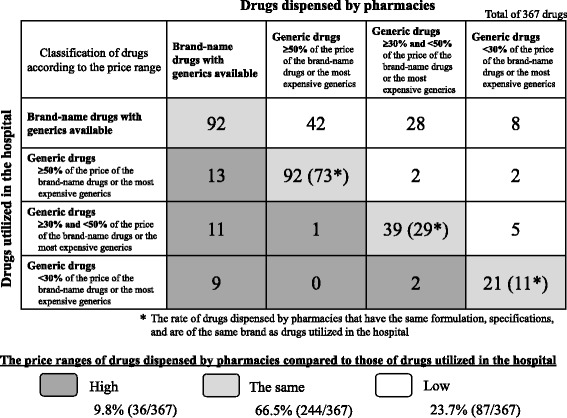
Price ranges of drugs dispensed by pharmacies versus those utilized in the hospital

The numbers of drugs for each price range are shown in Fig. 2. The percentages of drugs dispensed by pharmacies that were in higher price ranges, the same price range, and lower price ranges than drugs utilized in the hospital were 9.8% (36 drugs/367 drugs), 66.5% (244/367), and 23.7% (87/367), respectively. The rate of drugs dispensed by pharmacies that had the same formulation, specifications, and were of the same brand as drugs utilized in the hospital were 79.3% (73/92), 74.4% (29/39) and 52.4% (11/21) in the same ranges (≥50%, ≥30% and < 50% and < 30% of the price of the brand-name drugs or the most expensive generics), respectively.

## Discussion

In this study, we conducted assessments of the dispensation rates and costs of generic drugs and calculated cost reductions in order to uncover the effect that the adoption of generic drugs by a hospital has on the dispensation of generic drugs by community pharmacies. We found that both the dispensation rate and the price percentage of generic drugs dispensed by pharmacies increased significantly before and after the adoption of generic drugs by the hospital. As well, the numerical share of generic drugs in Japan was 46.7% in September 2013 and 56.2% in September 2014 [19]. Thus, there has been a trend toward the increased use of generic drugs in Japan.

In order to determine whether these observed significant increases in dispensation rates and price percentages of generic drugs were indeed affected by the adoption of generic drugs by Gifu Municipal Hospital, we performed two types of stratification: [1] stratification of drugs that had the same ingredients as the newly adopted generic drug vs. drugs that did not, and [2] stratification by prescribing facility. The dispensation rates and price percentages of generic drugs underwent large and significant increases for drugs with the same ingredients as the newly adopted generic drugs and drugs prescribed by Gifu Municipal Hospital, suggesting that the adoption of in-hospital use of generic drugs by Gifu Municipal Hospital promoted the dispensation of generic drugs by pharmacies.

According to a survey of the state of generic drug use by pharmacies, many health insurance pharmacies were not actively handling generic drugs owing to the fact that nearby medical facilities were not actively using generics [20]. It has also been reported that patients who had previously used generics had a greater desire to receive generic drugs from pharmacies than patients who had never used generics [9]. The most common reason given by patients for refusing generic drugs was “I don’t want to change from the drug I am currently using” [10]. In the present study, there were a couple of factors that may have explained the increases in dispensation rates and drug price percentages of generic drugs dispensed by pharmacies. First, local pharmacies had become more actively involved in the use of generic drugs as a result of the active adoption of generics by the hospital. Second, patients who had been admitted to the hospital had used generics while hospitalized and, as a result, chose to continue using generics at health insurance pharmacies even after being discharged from the hospital.

Our results found evidence of greater reductions in drug prices (per drug or per unit) dispensed by pharmacies after the adoption of generics. These cost reductions were particularly extensive for drugs prescribed by Gifu Municipal Hospital compared to drugs prescribed by other medical facilities. The stratification by Gifu Municipal and other hospitals/clinics were performed to exclude influencing factors such as other government efforts pushing the use of generic drugs and greater public awareness of generic drugs. Therefore, we believe this enhanced cost reduction occurred as a result of the increased number of generic drugs prescribed by Gifu Municipal Hospital. Based on the above data, the adoption of in-hospital use of generic drugs can be expected to increase the rate of generics used by pharmacies and can lead to cost reductions due to patients switching from brand-name drugs to generic drugs.

The current study also uncovered interesting findings regarding the price differences between drugs utilized in the hospital versus those dispensed by pharmacies. In particular, we found that 66.5% of drugs dispensed by pharmacies were in the same price range as those utilized in the hospital, which is a very high result. The rate of drugs dispensed by pharmacies that have the same formulation, specifications, and are of the same brand as drugs utilized in the hospital were higher than 50%, suggesting that it is easy for pharmacies to dispense the same generic drugs (the same formulation, specifications and brand) that are used in the hospital. Only 9.8% of drugs dispensed by pharmacies were in a higher price range, indicating that pharmacies were actively dispensing generics.

The limitations of this study include the fact that it was a retrospective study conducted at a single general hospital in a single community, as well as the fact that it surveyed drugs used by patients at the time of their admission. Furthermore, there might be several other sources of potential bias. In Japan, drugs that are prescribed by hospitals and clinics are often dispensed by pharmacies located near those medical facilities. Clinics play a role in the function of “home doctor” and most of hospitals play a central role in community medical care. Because most patients in the community go to the hospital central in the community, we believe that the use of in-hospital patients at a single general hospital in a single community was appropriate in considering the background of the Japanese medical system.

## Conclusions

The current study suggests that the use of generic drugs by pharmacies is promoted and drug costs are lowered as a result of the adoption of generic drugs by a large hospital. The data in this study constitutes important evidence that can be used by the national government to create proposals to reform the healthcare system and revise government medical policies.

## References

[1] IMS-Health, MIDAS, Market Segmentation, MAT Sep 2015, RX only (Prescription Bound). 2015.

[2] Ministry of Health, Labour and Welfare (Japan). http://www.mhlw.go.jp/bunya/iryouhoken/database/ (2015) Accessed 5 Feb 2018.

[3] Ministry of Health, Labour and Welfare (Japan). Roadmap to further promote the use of generics. http://www.mhlw.go.jp/stf/shingi/2r9852000002yu25-att/2r9852000002zb0m_1.pdf (2013) Accessed 5 Feb 2018.

[4] Hassali MA, Shafie AA, Jamshed S, Ibrahim MI, Awaisu A (2009). Consumers’ views on generic medicines: a review of the literature. Int J Pharm Pract.

[5] Hassali MA, Wong ZY, Alrasheedy AA, Saleem F, Mohamad Yahaya AH, Aljadhey H (2014). Perspectives of physicians practicing in low and middle income countries towards generic medicines: a narrative review. Health Policy.

[6] Toverud EL, Hartmann K, Hakonsen H (2015). A Systematic Review of Physicians’ and Pharmacists’ perspectives on generic drug use: what are the global challenges?. Appl Health Econ Health Policy.

[7] Dunne SS, Dunne CP (2015). What do people really think of generic medicines? A systematic review and critical appraisal of literature on stakeholder perceptions of generic drugs. BMC Med.

[8] Colgan S, Faasse K, Martin LR, Stephens MH, Grey A, Petrie KJ (2015). Perceptions of generic medication in the general population, doctors and pharmacists: a systematic review. BMJ Open.

[9] Kobayashi E, Karigome H, Sakurada T, Satoh N, Ueda S (2011). Patients’ attitudes towards generic drug substitution in Japan. Health Policy.

[10] Fukumoto K, Oishi M, Ohkubo A, Ohkubo K, Saitoh M, Kurihara K (2009). A questionnaire survey of pharmacist and patient attitudes towards generic drugs. Jpn. J Generic Med.

[11] Tanaka H, Sato T, Maeda M (2002). Investigation and its analysis of attitude toward substitute dispensation using generic medicine among general practitioners. Jpn J Pharm Care Sci.

[12] Sakurai H, Itoh Y, Hashizume K, Yamaguchi T, Yoshimachi S, Sugiyama H (2011). Survey of patients attitudes toward generic drug substitution in community pharmacies. Jpn J drug Inform.

[13] Nagai N, Ono H, Yamato M, Horino T, Kitakouji M, Ito Y (2012). An investigation on attitudes toward the promotion of generic products for pharmacists in chain community pharmacies in Japan. Jpn J Pharm Care Sci.

[14] Howard JN, Harris I, Frank G, Kiptanui Z, Qian J, Hansen R. Influencers of generic drug utilization: a systematic review. Res Social Adm Pharm. 2017; 10.1016/j.sapharm.2017.08.001. [Epub ahead of print] 10.1016/j.sapharm.2017.08.001 PMC5910277 28814375

[15] Tachi T, Saito K, Esaki H, Kanematsu Y, Yoshida A, Sugita I, et al. Medical and economic factors influencing generic drug use in the Japanese public health system: influencing factors in different populations. Int J Health Plann Manag. 2018; 10.1002/hpm.2489. [Epub ahead of print] 10.1002/hpm.2489 29315859

[16] Akase T, Tsuchiya T, Kobayashi M (2015). Study on lengths of stay and use of pharmaceutical products on DPC date in a hospital group. J Assoc Health Care Admin.

[17] Kawai M, Tajiri C, Kato T, Takeuchi Y (2011). Economic Benefits and Problems concerning the introduction of generic drugs in a hospital with a DPC system. Jpn J Generic Med.

[18] Ministry of Health, Labour and Welfare (Japan). http://www.mhlw.go.jp/file/05-Shingikai-12404000-Hokenkyoku-Iryouka/0000166044.pdf (2017) Accessed 5 Feb 2018.

[19] Ministry of Health, Labour and Welfare (Japan). http://www.mhlw.go.jp/stf/seisakunitsuite/bunya/kenkou_iryou/iryou/kouhatu-iyaku/ (2014) Accessed 5 Feb 2018.

[20] Ministry of Health, Labour and Welfare (Japan). http://www.mhlw.go.jp/file/05-Shingikai-12404000-Hokenkyoku-Iryouka/0000049161.pdf (2014) Accessed 5 Feb 2018.

